# Reresection of Colorectal Liver Secondaries: A Preliminary Report

**DOI:** 10.1155/1990/81713

**Published:** 1990

**Authors:** Roland Andersson, Karl-Göran Tranberg, Stig Bengmark

**Affiliations:** 1 Department of Surgery Lund University Lund Sweden; 2 Department of Surgery Lund University Lund S-221 85 Sweden

## Abstract

During a 4.5–year period, 5 patients underwent reresection of colorectal liver metastases. Two patients died of recurrent disease, 9–11 months after reresection. Three patients are alive, one without and two with recurrent disease, 15, 15 and 68 months after reresection. Although our results suggest that liver reresection may be meaningful in selected patients with colorectal liver metastases, further studies are necessary in order to define candidates for this procedure.


*HPB Surgery*, 1990, Vol. 2, pp. 69–72
Reprints available directly from the publisher
Photocopying permitted by license only

© 1990 Harwood Academic Publishers GmbH
Printed in the United Kingdom

### CASE REPORTS

### RERESECTION OF COLORECTAL LIVER SECONDARIES: A PRELIMINARY REPORT

ROLAND ANDERSSON*, KARL-GÖRAN TRANBERG and STIG BENGMARK

*Department of Surgery, Lund University, Lund, Sweden*

*(Received 15 May 1989)*

During a 4.5–year period, 5 patients underwent reresection of colorectal liver metastases. Two patients died of recurrent disease, 9–11 months after reresection. Three patients are alive, one without and two with recurrent disease, 15, 15 and 68 months after reresection. Although our results suggest that liver reresection may be meaningful in selected patients with colorectal liver metastases, further studies are necessary in order to define candidates for this procedure.

KEY WORDS: Colorectal liver cancer, surgery, resection

### INTRODUCTION

Liver resection gives 5-year survival rates of about 30% in both primary colorectal and primary liver cancer[1,2]. Except for dearterialization procedures for carcinoid liver disease, and liver transplantation for primary liver cancer[3,4], no other treatment has been demonstrated to achieve cure or long-term palliation in malignant liver disease.

Treatment of recurrent cancer after liver resection is usually palliative and may include chemotherapy. Relapse after resection of colorectal liver metastases is usually not confined to a single site; the liver, lungs and peritoneal cavity being the most common sites of first recurrence. It appears, however, that about 1/3 of patients with relapse have (the initial) relapse in the liver alone, indicating a role for repeat liver resection in selected patients[5].

This paper reports our preliminary experience of reresection of colorectal liver metastases and tries to answer whether continued evaluation of such aggressive efforts is justified.

### PATIENTS

From July 1983 to December 1987, 5 patients, 2 female and 3 male, underwent reresection of colorectal liver cancer. The mean age at reresection was 57 (range: 44–70) years. Synchronous metastatic disease in the liver was detected at the

* Correspondence to: Roland Andersson, M.D., Department of Surgery, Lund University, S–221 85 Lund, Sweden.

primary abdominoperineal resection for rectal carcinoma (Dukes C) in 1 patient and at left hemicolectomy (Dukes C) in 1; metachronous disease was detected 4 months, 1 year and 4 years after left hemicolectomy for sigmoid carcinoma (Dukes C, C and A, respectively) (Table 1).

**Table 1** Patients.

| Patient no. | Primary tumor | Age (atresection) y | Sex | 1st liver resection: months after primary op | no. of metastases | type of operation |
|---|---|---|---|---|---|---|
| 1 | rectal cancer (Dukes C) | 70 | M | 3 | 1 | right lobectomy |
| 2 | sigmoid cancer (Dukes C) | 44 | M | 12 | 1 | right lobectomy |
| 3 | sigmoid cancer (Dukes A) | 61 | F | 48 | 1 | right lobectomy |
| 4 | sigmoid cancer (Dukes C) | 51 | F | 2 | 1 | resection of quadrate lobe |
| 5 | sigmoid cancer | 62 | M | 4 | 1 | right lobectomy |

A solitary liver deposit was removed by right hepatectomy in 4 patients, 3–48 months after the primary operation, and by resection of the quadrate lobe in 1 patient, 2 months after the primary operation. Resection margins were free of tumour in all resected specimens. Intraoperative ultrasonography was not available.

### RERESECTION OF THE LIVER

Recurrent disease in the liver was asymptomatic and detected at routine follow-up that included physical examination, liver function tests and computed tomography. The interval between the first and second liver resections varied between 4 and 37 months with a median interval of 6 months (Table 2).

Wedge resections or larger, "atypical", resections were performed in all patients. The number of liver secondaries varied from 1–10; three patients had single wedge or atypical resections and two patients had an atypical resection combined with a wedge resection or resection of the caudate lobe.

### RESULTS

The results are summarized in Table 2. Three patients are alive; one of them has no evidence of disease 15 months after reresection (as estimated by liver function tests, carcinoembryonic antigen levels, pulmonary x-ray and ultrasound of the liver); the other two patients have relapse in the lungs (both patients) and the liver (one patient) at 15 and 68 months after reresection. Two patients died of recurrent disease, in the liver or skeleton, 9 and 11 months after reresection. The (cumulative) number of liver metastases appeared to have prognostic significance: of the two patients with a solitary metastasis at both liver resections, one is alive without evidence of disease whereas the other has survived for 68 months after reresection but with evidence of relapse in the liver and the lungs.

Postoperatively, one patient had a subhepatic abscess requiring surgical drainage.

**Table 2** Reresection of liver metastases.

| Patient no. | Months after 1st liver resection | No. of metastases | Type of resection | Morbidity | Recurrence | Survival (after reresection), mo |
|---|---|---|---|---|---|---|
| 1 | 4 | 10 | Atypical | | liver, peritoneal cavity | 11 |
| 2 | 37 | 2 | Atypical + wedge | Subhepatic abscess | skeleton | 9 |
| 3 | 5 | 1 | Atypical | | liver, lungs | 68(LWD) |
| 4 | 7 | 1 | Wedge | | 0 | 15(NED) |
| 5 | 6 | 3 | Atypical + caudate lobe | | lungs | 15(LWD) |

LWD = living with disease
NED = no evidence of disease

### DISCUSSION

Information about the value of reresection of liver cancer is scanty and can be found only in a few recent reports. As for hepatocellular carcinoma, Nagasue *et al.* and Kanematsu *et al.* reported that a second hepatic resection was followed by meaningful palliation in some patients and, probably, survival benefit in a few[6,7]. As for liver metastases, Nordlinger *et al.* reported the fate of 6 patients having undergone reresection for colorectal liver cancer: 2 patients were alive without evidence of disease after 2 and 12 months, 3 patients were alive with recurrent disease after 14, 16 and 28 months, and 1 patient died 10 months following reresection[8]. A similar experience was reported also by Fortner[9].

In our study, it appears that the two patients with more than one deposit at reresection did not benefit from the procedure. On the other hand, it appears likely that reresection was beneficial in the two patients having single deposits at both the first and second liver resection and thus a cumulative number of 2 liver tumours.

For colorectal cancer, previous studies have indicated that (a first) liver resection is indicated when (a) the number of liver tumors is 3 or less, (b), extrahepatic tumor is not demonstrable, and (c) a resection margin of at least 10 mm can be obtained[10,11]. Although the review of the literature and our own results indicate that few patients benefit from reresection of colorectal liver secondaries, our data suggest that reresection is worth a try in patients having a cumulative number of 2

liver metastases. However, further experience is needed for proper definition of reresection criteria.

The fact that four of the five patients have developed recurrent disease (hepatic recurrence in 2) emphasizes the inadequacy of presently available diagnostic methods (Table 2). Since 4 patients developed hepatic relapse within 7 months after the first liver resection, it should be noted that these recurrences did not occur at the site of the initial resection but were missed at the first operation. Anyhow, these early recurrences suggest that intraoperative ultrasonography should have been helpful in detecting these "occult" metastases at the first hepatic resection. The use of intraoperative ultrasonography, now available at our and other centres, would lead to a better selection of patients for liver resection and, possibly, improved criteria for liver (re)resection.

Since the two patients with hepatic recurrence also had extrahepatic relapse, none of them would have been helped with liver transplantation. At any rate, the dismal experience with liver transplantation for "isolated" colorectal liver cancer[4,12,13] contraindicates liver grafting in patients with colorectal liver metastases.

### *References*

1. Adson, M.A. (1986) Liver resection in primary and secondary liver cancer. Clinical Surgery International, vol. 12, Bengmark S., Blumgart L.H. eds. *Liver Surgery*. London: Churchill Livingstone, 51–62.
2. Bengmark, S., Ekberg, H. and Tranberg, K.-G. (1987) Metastatic tumours of the liver. In: Blumgart L.H. ed. Surgery of the Liver and the Biliary Tract. London: Churchill Livingstone, 1179–1189.
3. Scharschmidt, B.F. (1984) Human liver transplantation: analysis of data on 540 patients from four centers. *Hepatology*, **4**, 95S--101S.
4. Polson, R.J., O'Grady, J.G. and Williams, R. (1987) Liver transplantation in the treatment of hepatic malignancies. Bailleres Clin. *Gastroenterol.*, **1**, 171–182.
5. Ekberg, H., Tranberg, K.-G., Andersson, R., Lundstedt, C., Hägerstrand, I., Ranstam, J. and Bengmark, S. (1987) Pattern of recurrence in liver resection for colorectal secondaries. *World J. Surg.*, **11**, 541–547.
6. Nagasue, N., Yukaya, H., Ogawa, Y., Sasaki, Y., Chang, Y.-C. and Niimi, K. (1986) Second hepatic resection for recurrent hepatocellular carcinoma. *Br. J. Surg.*, **73**, 434–438.
7. Kanematsu, T., Matsumata, T., Takenaka, K., Yoshida, Y., Higashi, H. and Sugimachi, K. (1988) Clinical management of recurrent hepatocellular carcinoma after primary resection. *Br. J. Surg.*, **75**, 203–206.
8. Nordlinger, B., Parc, R., Delva, E., Quilichini, M.-A., Hannoun, L. and Huguet, C. (1987) Hepatic resection for colorectal liver metastases: influence on survival of preoperative factors and surgery for recurrences in 80 patients. *Ann. Surg.*, **205**, 256–263.
9. Fortner, J.G. (1988) Recurrence of colorectal cancer after hepatic resection. *Am. J. Surg.*, **155**, 378–382.
10. Ekberg, H., Tranberg, K.-G., Andersson, R., Lundstedt, C., Hägerstrand, I., Ranstam, J. and Bengmark, S. (1987) Determinants of survival in liver resection for colorectal secondaries. *Br. J. Surg.*, **73**, 727–731.
11. Hughes, K.S. and the Registry of Hepatic Metastases. (1986) Resection of the liver for colorectal carcinoma metastases: a multi-institutional study of patterns of recurrence. *Surgery*, **100**, 278–284.
12. Pichlmayr, R., Ringe, B., Lauchart, W. and Wonigeit, K. (1987) Liver transplantation. *Transplant. Proc.*, **19**, 103–112.
13. Muhlbacher, F. and Piza, F. (1987) Orthotopic liver transplantation for secondary malignancies of the liver. *Transplant. Proc.*, **19**, 2396–2398.

*(Accepted by L. Blumgart 28 June 1989)*

